# PAM trial protocol: a randomised feasibility study of psychedelic microdosing–assisted meaning-centred psychotherapy in advanced stage cancer patients

**DOI:** 10.1186/s40814-024-01449-9

**Published:** 2024-02-12

**Authors:** Alesha Wells, A. P. Suresh Muthukumaraswamy, Eva Morunga, Will Evans, Alana Cavadino, Mahima Bansal, Nicola J. Lawrence, Amanda Ashley, Nicholas R. Hoeh, Frederick Sundram, Allison J. Applebaum, Hineatua Parkinson, Lisa Reynolds

**Affiliations:** 1 Department of Psychological Medicine, Faculty of Medical and Health Sciences, University of Auckland, 22-30 Park Avenue, Grafton, Auckland, 1023 New Zealand; 2 School of Pharmacy, Faculty of Medical and Health Sciences, University of Auckland, 85 Park Road, Grafton, Auckland, 1023 New Zealand; 3 Cancer Support Service, Te Toka Tumai Auckland, Auckland City Hospital, Te Whatu Ora2 Park Road, Grafton, Auckland, 1023 New Zealand; 4 The University of Auckland, 22-30 Park Avenue, Grafton, Auckland, 1023 New Zealand; 5 Mana Health, 7 Ruskin Street, Parnell, Auckland, 1052 New Zealand; 6 School of Population Health, The University of Auckland, 22-30 Park Avenue, Grafton, Auckland, 1023 New Zealand; 7 Department of Oncology, Faculty of Medical and Health Sciences, University of Auckland, 85 Park Road, Grafton, Auckland, New Zealand; 8 Te Pūriri o Te Ora – Regional Cancer and Blood, Te Whatu Ora Te Toka Tumai, 2 Park Road, Grafton, Auckland, 1023 New Zealand; 9 Harbour Cancer and Wellness, 212 Wairau Road, Wairau Valley, Auckland, 0627 New Zealand; 10 Department of Psychiatry and Behavioral Sciences, Memorial Sloan Kettering Cancer Center, 641 Lexington Avenue, 7th Floor, New York, NY 10022 USA; 11 School of Psychology, University of Auckland, 23 Symonds Street, Auckland Central, 1010 New Zealand

**Keywords:** Microdosing, Lysergic acid diethylamide, Psychedelics, Cancer, End-of-life distress, Meaning-centred psychotherapy

## Abstract

**Background:**

An advanced cancer diagnosis can be associated with a significant profile of distress. Psychedelic compounds have shown clinically significant effects in the treatment of psychological distress in patients with advanced-stage cancer. Given the challenges of delivering timely and effective intervention in the advanced cancer context, it is possible that an alternative, more pragmatic, approach lies in psychedelic ‘microdosing’. Microdosing refers to repeated administration of psychedelics in sub-hallucinogenic doses. The purpose of this study is to evaluate the feasibility of conducting a full-scale randomised controlled trial comparing psychedelic microdose-assisted–meaning-centred psychotherapy (PA-MCP) to standard meaning-centred psychotherapy (MCP) in New Zealand indigenous (Māori) and non-indigenous people with advanced cancer and symptoms of anxiety and/or depression. Although MCP is a well-established psychotherapeutic treatment in advanced cancer populations, the potential efficacy and effectiveness of this therapy when delivered alongside a standardised microdose regimen of a psychedelic compound have not been investigated.

**Methods:**

Participants with advanced-stage cancer and symptoms of anxiety and/or depression (*N* = 40; 20 Māori, 20 non-Māori) will be randomised under double-blind conditions to receive 7 sessions of MCP alongside 13 doses of either an LSD microdose (4–20 µg) (PA–MCP) or inactive placebo (placebo-MCP). The feasibility, acceptability, and safety of this intervention and physiological and psychological measures will be recorded at baseline, at each session of MCP, and at a 1-month and 6-month follow-up.

**Discussion:**

Our findings will evaluate the feasibility, acceptability, and safety of a larger randomised controlled trial and provide an initial indication of the potential benefits of psychedelic microdosing for psychological distress in advanced-stage indigenous and non-indigenous cancer patients.

**Trial Registration:**

NZCTR, ACTRN12623000478617. Registered 11 May 2023. https://www.anzctr.org.au/Trial/Registration/TrialReview.aspx?id=385810&isReview=true.

**Supplementary Information:**

The online version contains supplementary material available at 10.1186/s40814-024-01449-9.

## Background and rationale

Patients with cancer often develop clinically significant symptoms of psychological distress. In particular, people with advanced-stage cancer have a high prevalence of depression, anxiety, and reduced quality of life, with 40% meeting the criteria for a mood disorder [1–3]. Such disorders can significantly impact a patient’s end-of-life experience, contributing to feelings of loss of meaning, demoralisation, and a desire for hastened death (otherwise known as ‘existential distress’). Such impacts represent a significant challenge in palliative medicine [4, 5]. Additionally, depression and anxiety have been associated with decreased treatment adherence [6], prolonged hospitalisation [7], decreased quality of life [6], and increased suicidality [8] in this population. Depression is an independent risk factor for early death in cancer patients [6, 9].

The efficacy of standard treatment approaches to anxiety and depression in cancer patients is mixed and limited [1, 10]. Pharmacotherapeutic interventions are commonly used to treat anxiety and depression in this context; however, they have notable limitations [1, 10]. Several meta-analyses of placebo-controlled trials of antidepressants have failed to demonstrate a clear effect of treatment in cancer patients [11–13]. The onset of clinical improvement with antidepressants in cancer is delayed, relapse rates are high, and significant side effects compromise treatment adherence [14]. Likewise, practical barriers often limit the feasibility of psychological interventions in cancer, especially in advanced stages, when patients face considerable symptom load and burden from treatment and medical commitments [15, 16]. Low participation and high attrition of psychological therapies are common; thus, psychosocial interventions in this context need to be timely, brief, and effective. The need to develop alternative and effective therapeutic approaches to mitigate the negative effects of advanced cancer has become increasingly recognised within the disciplines of palliative care and psycho-oncology.

### Meaning-centred psychotherapy in people with advanced cancer

Meaning-centred psychotherapy (MCP) was developed in response to the despair, hopelessness, loss of meaning, and desire for hastened death commonly occurring in people with advanced cancer, i.e. where a cancer diagnosis is unlikely to be controlled or cured with treatment [17]. A recent systematic review of psychosocial interventions with advanced cancer patients noted that there is compelling evidence for using MCP to improve meaning and quality of life in this population [18]. MCP is a psychological intervention tailored to the needs of patients with a life-limiting cancer diagnosis and is influenced by the work of psychiatrist Viktor Frankl [19]. MCP is an existential therapeutic approach that combines didactic components, discussion, and experiential exercises to facilitate participants’ understanding and connection to various sources of meaning [20]. The goal of MCP is to support patients’ understanding of the concept of meaning and its importance in life, particularly as they face the ultimate limitation of impending death.

Although initially developed as a group intervention, MCP has since been adapted for individuals to increase the flexibility of treatment implementation because scheduling or illness-related problems can hinder attendance in a group. Individualised MCP follows a seven-session protocol, with each session focussing on a specific theme related to exploring sources of meaning and purpose in life [21, 22]. These seven sessions are best delivered weekly over 7 weeks; however, due to the characteristics of the target population, the standardised protocol contains contingencies for a decline in wellness or competing health commitments. The upper range of per-protocol delivery is therefore 7 sessions over 14 weeks. This therapeutic approach has been shown to reduce anxiety and a desire for hastened death and improve spiritual well-being, meaning, and overall quality of life in patients with advanced cancer [22]. A recent systematic review of psychosocial interventions with advanced cancer patients noted that there is compelling evidence for the use of MCP to improve quality of life in this population [18]. Nevertheless, effect sizes of MCP compared to usual care are only small to medium (*d* = 0.1 to 0.34) [22]. Evidence that pharmacological interventions can serve as adjuncts to psychotherapy [23] raise the possibility that the benefits of MCP might be both enhanced and expedited if delivered in conjunction with a pharmacological approach.

### Potential of psychedelics

Psychedelic compounds offer a promising pharmacological approach in the context of advanced cancer. Classical psychedelics, which include psilocybin (psilocin) and lysergic acid diethylamide (LSD), are a structurally diverse group of compounds that are 5-HT_2A_ receptor agonists and produce a unique profile of changes in thoughts, perceptions, and emotions [24, 25]. Several unblinded studies in the 1960s and 1970s suggested that such compounds might effectively treat psychological distress in cancer patients [26–28]; however, these studies did not include the comparison conditions expected of modern, rigorous psychopharmacology trials. Subsequently, human research with these compounds was halted for almost three decades because of politically charged concerns regarding safety resulting in most psychedelic compounds being classified as Schedule 1, ‘drugs of abuse’. The recent resumption of clinical research investigating the therapeutic potential of psychedelic agents in the USA, Australia, and Europe has established conditions for the safe administration and use of these drugs [29, 30]. Research specifically with advanced cancer participants has demonstrated no serious medical or psychiatric adverse effects [31, 32].

Emerging work investigating the utility of psychedelic compounds (e.g. LSD, psilocybin) alongside psychotherapy in the context of cancer-related anxiety and depression suggests a novel approach worthy of consideration. A review of clinical trials in cancer settings demonstrated that guided psychedelic experience alongside psychological therapy could produce rapid, robust, and sustained improvements in cancer-related psychological distress [33]. One recent study of note from Johns Hopkins University found that psychotherapy in conjunction with a single high dose of a psychedelic compound (psilocybin) to induce a hallucinogenic experience produced sustained reductions in existential distress (depression, anxiety, and fear of death) and increases in quality of life in cancer patients who had long-standing symptoms of depression and/or anxiety [31]. A similar study conducted with cancer patients experiencing cancer-related anxiety or depression produced immediate and sustained improvements in anxiety and depression following a single high dose of psilocybin combined with psychotherapy [32]. Participants also reported reductions in demoralisation, and hopelessness, and improved spiritual well-being and quality of life, effects that persisted at the 6.5-month follow-up. Such fast-acting and effective responses are particularly important in the context of a life-limiting illness where timeliness is a priority. However, there are considerable barriers to high-dose psychedelic intervention in the advanced cancer context insofar that patients are extremely vulnerable and often have numerous complex and competing personal and medical commitments. Additionally, patients may be reluctant to participate in an intervention where high doses of psychedelics have been associated with transient episodes of psychological distress.

### Microdosing of LSD

It is possible that an alternative, more pragmatic, approach to high-dose psychedelic administration lies in psychedelic ‘microdosing’, i.e. low doses of psychedelics that do not elicit hallucinogenic effects but potentially enable positive effects such as relaxation, creativity, and openness to new ideas. Qualitative and observational studies suggest that microdosing might improve mood and counteract symptoms of anxiety and depression in much the same way that larger doses have been found to do [34]. Recent double-blind, placebo-controlled experiments of LSD microdosing have indicated increases in the neurotrophic factor BDNF related to cortical plasticity [35]; increases in pain tolerance [36]; changes in perceptions of time [37]; improvements to attention [38]; and acute increases in self-ratings of creativity, connectedness, energy, happiness, irritability, and wellness [39]. Given these short-term positive improvements in feelings of mood, it is possible that microdosing alongside psychological therapy might facilitate and, perhaps, expedite therapeutic change. However, whilst microdosing is a growing trend in popular culture [40], there are no controlled scientific studies of the effects of psychedelic microdosing in cancer.

The present study aims to address this gap and builds on the recently completed Phase 1 LSD microdosing trial conducted at The University of Auckland (MDLSD study), which investigated the effects of microdosing LSD in a healthy volunteer population. The proposed study will follow a similar approach and microdosing regimen [39]. Briefly, participants will be randomised to receive either placebo or a microdose of LSD two times a week alongside an evidence-based psychotherapy (see below) for 6 weeks and 1 day. Participants will receive a total of 13 doses across the study duration.

### Safety and tolerability

Despite the good safety profile of LSD [40], surveys of psychedelic microdosers often report mild side effects. These include psychological effects, such as racing thoughts or increased anxiety, and physiological effects, such as headaches or sleep problems [41]. However, not everyone who microdoses report side effects. Among an online sample of psychedelic microdosers, 30% reported no side effects [41], and in another online study of 1116 microdosers, only 20% of the sample reported adverse effects [42]. Furthermore, these effects are reported to be acute rather than persisting long term. However, results from observational research need to be verified in more scientifically rigorous trials, as variability in microdosing practices, dosages, quality of substances, and measurement time points limit the validity of the results.

To date, several laboratory-based randomised controlled trials have explored the effects and safety of LSD microdosing. Excluding the MDLSD study [39] (see below), five studies with unique samples have been conducted. Safety data collected in these trials consisted primarily of blood pressure, heart rate, and basal body temperature. The first tested three doses of LSD tartrate (6.5, 13, 26 μg) compared to placebo in a within-subjects design [43]. Participants in this study reported dose-dependent increases on the 5D-ASC, a scale used to measure consciousness-altering effects. An increase in blood pressure, but not body temperature or heart rate, was observed at the 13 and 26 μg doses, an effect observed in other research [38, 44]. No other safety data or adverse events were reported. In another study, the highest dose produced modest subjective effects, including increased ratings of ‘feeling a drug effect’, significant peak change scores on the Profile of Mood States Vigor subscale, and ratings of stimulant-like and LSD-like effects [45]. However, no effects on any cardiovascular measures were reported.

The final of these five studies reported more detailed treatment-emergent adverse events (TEAEs) in 48 healthy older volunteers receiving six doses, 1 every 3 days, of either 0, 5, 10, or 20 μg of LSD [46]. Although between 66.7 and 83.3% of participants in each group reported TEAEs, the only statistically significant difference between groups was the frequency of headaches. The percentage of volunteers at LSD doses 5 μg, 10 μg, and 20 μg reporting headaches were 16.7%, 50.0%, and 25.0% respectively compared to 8.3% in the placebo group. All headaches were either mild or moderate. The frequency and severity of all other adverse events were not different from placebo. Furthermore, no adverse events were severe in intensity, with no unexpected adverse events being reported. Vital signs, physical examinations, ECG, and laboratory results produced no clinically significant abnormalities [46].

Results of safety data from the MDLSD study [39] showed a favourable safety profile with no changes in vital signs, and this study did not replicate the previous report of increased headaches. The most notable adverse effect in the MDLSD trial was that 10% of participants reported increased levels of anxiety leading to discontinuation. In the MDLSD trial, a dose titration protocol was introduced mid-trial to attempt to reduce anxiety—an approach that successfully improved the retention of participants in the trial.

### Scientific basis for current study design

The current microdosing protocol dose range considers three key factors: (1) common community microdosing practices, (2) the recent MDLSD protocol [41], and (3) the needs of the study population. The MDLSD dosing protocol (10 μg dose, every third day) is based on the community practice of taking approximately 10% of a standard recreational dose, in alignment with the popular schedule outlined by Fadiman [47, 48]. The MDLSD dosing schedule has been adapted in the current work to better reflect the requirements of the study population in three ways. These adaptations include a reduced starting dose (8 μg), the introduction of dose titration, and a reduction in dose frequency. A patient population nearing end-of-life is expected to have higher anxiety at baseline compared to a healthy population; they may also be undergoing non-curative treatment at the time of intervention. This modified dosing protocol aims to reduce the likelihood of an initial anxiety response to the first dose, allowing participants to titrate up or down (between 4 and 20 μg) based on their response. The reduced dosage frequency, from every third day to two times per week, allows more flexibility for the participants to select a dosing day that works with their lifestyle and other medical commitments. One of the weekly doses will align with the MCP session, whilst the second dose will ideally be on a day of the week when they can engage in MCP homework activities, with the aim of using these home doses to compound the effects of the therapy.

Previous research has highlighted the role of expectancy effects in the use and effects of psychedelics. In a sample of 81 healthy participants who engaged in microdosing with psychedelics, the researchers found that expectancy scores at baseline were significantly associated with improvements in well-being [49]. Much of the research conducted on microdosing has been observational research looking at existing microdosers, making it more challenging to separate the role of expectancy/placebo effects and the actual effect of psychedelics [50–53]. Many studies of psychedelic drugs purport by design to be double or single blind. However, the powerful psychological effects of these drugs effectively unblind participants which can potentially bias effect size estimates [54]. Data from the MDLSD study [39] indicate that microdoses of LSD can lead to functional unblinding of participants—particularly those in the active group—and, as such, extra study design features are needed to try to better maintain blinding. As a result, the current protocol opts for the use of mild deception. Participants will be told that one of three placebos will be used: inactive placebo, caffeine, or methylphenidate. The psychological effects of these substances reasonably approximate the side effects reported in other microdosing studies. Participants will be informed that the placebo allocation ratio is 1/3:1/3:1/3 when it is in fact 1:0:0 for inactive placebo, caffeine, and methylphenidate, respectively. Such mild deception has been used effectively in similar studies previously [45].

Prior to trial commencement, an open-label drug-free sub-study was conducted with 6 participants. This sub-study utilised the same study protocols outlined in this article, excluding any protocol aspects related to drug administration or the measurement of drug effects. The purpose of the PAM Trial sub-study was to create an opportunity for research staff to gain experience in delivering the protocol and finetune study delivery before integrating the investigational medicinal product (IMP) component of the PAM Trial. The process of refinement was based on qualitative participant feedback and research staff experience. Importantly, the sub-study provided the trial psychologists an opportunity to familiarise themselves with the study design and develop their MCP therapist skills.

### Cultural considerations

In New Zealand, an important part of the research design process is ensuring research is culturally responsive to our nation’s indigenous Māori peoples. Te Tiriti o Waitangi (The Treaty of Waitangi) outlines key principles that can be applied to the health research process. These principles are intricately reflected in the current study protocol. A driving force behind this research is achieving equitable health outcomes for Māori. Within the context of cancer, Māori experience significant health disparities. The rate of cancer diagnoses and cancer mortality is disproportionally higher for Māori compared to non-Māori (Ministry of Health, 2018). Furthermore, Māori are 1.5 times more likely than non-Māori to report an anxiety or depressive disorder (Ministry of Health, 2014). Such disparities reinforce the importance that Māori are included at all stages in developing cancer-related psychological interventions so that Māori benefit from any interventions aimed at improving outcomes. This includes Māori consultation in research design, involvement of Māori researchers, advisors, and psychologists, and importantly the equal recruitment of Māori and non-Māori participants. Aspects of Te Ao Māori (the Māori worldview) have also been integrated into the study assessments and delivery. For instance, holistic aspects of well-being according to Te Whare Tapa Wha domains (Durie, 1985) and wairua (spirituality; Barnes, Gunn, Barnes, Muriwai, Wetherell & McCreanor, 2017) will be assessed using measures that reflect these broad conceptualisations of health (FACIT–Spiritual Well-Being scale, Hua Oranga). Participants will also be encouraged to bring whānau (family) support to assessment and clinical sessions, and we will include the perspective of whānau in our analyses. Furthermore, all members of the research team will be appropriately aware of relevant tikanga Māori (customary practices). It is through these methods and an ongoing process of consultation that this research aims to bring benefit to our Māori community, with the ultimate aim of achieving equitable outcomes for Māori.

### Objectives

The primary objectives of the current study are to examine the feasibility, acceptability, and safety of a randomised, double-blind, placebo-controlled trial comparing PA-MCP to MCP with placebo in Māori and non-Māori patients with advanced-stage cancer.

In order to gain an initial indication of clinically meaningful change and generate data for sample size calculations of a larger trial, several secondary measures are included. These will assess the following constructs: sense of meaning, quality of life, spiritual well-being, demoralisation, attitudes towards death, anxiety, depression, and pain (for detail on scales see Table 5).

## Methods/design

### Study design

**Fig. 1:**
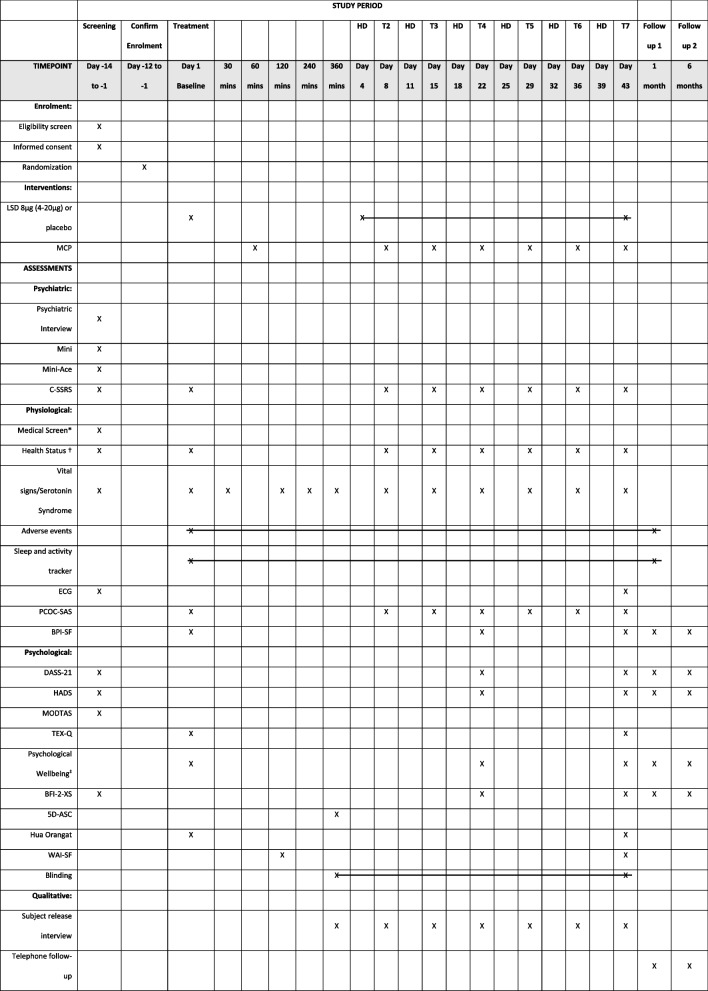
Schedule of enrolment, interventions, and assessments (abbreviated) NB: HD = Home dosing, *Medical History, Height, Weight, and Blood test, † AKPS, Change in Medication, Health Status Review, ‡ LAP-R-PMI, FACIT-SP-12, Demoralisation Scale, SAHD, and WCS

This study is a randomised, double-blind, placebo-controlled parallel group feasibility trial. The study will take place at the Clinical Research Centre in Grafton Campus of Auckland University in New Zealand. The study received ethics approval from the Health and Disability Ethics Committee (HDEC) on the 14th of July 2022 (Reference: 13,074). Eligible participants (*N* = 40; 20 Māori, 20 non-Māori) will be randomly allocated to one of two treatment groups (1) PA-MCP (*n* = 20) or (2) placebo with MCP (*n* = 20) (see Fig. 1). Participants in the PA-MCP condition will receive a treatment course of LSD microdoses starting at 8 μg (titration range: 4–20 µg); participants in the placebo condition will receive an inert substance identical in appearance (see the ‘Drug preparation and administration’ section).

Participants will be referred by regional cancer services or contact the study team directly in response to study advertisements.

Participants will all have a diagnosis of stage IV solid tumour cancer and moderate to severe distress, as identified by our screening questionnaire [55]. Half of the sample will identify as Māori, and the other half will identify as non-Māori. Participants will be required to meet the full inclusion and exclusion criteria outlined in Tables 1 and 2. Initial screening phone calls will be conducted with participants to explain study participation and inclusion/exclusion criteria and to establish the current level of distress on a scale from 1 to 10 (distress thermometer [55]).

**Table 1:** Full inclusion criteria

	Inclusion criteria
Consent	Willing and able to give informed consent for participation in the trial, reconfirmed verbally at each clinic visit
	Agree to have study visits video and/or audio recorded
	Agree to inform the Investigators within 48 h of any medical conditions and procedures being undertaken
	Willing for the investigators to communicate directly with their medical team to determine medical suitability for study participation (oncologist, GP, palliative care physician, etc.)
	Agree to refrain from starting any new psychiatric medication and/or psychotherapy during the study period
	Agree to have transportation other than driving themselves to where they are staying on the days of medication dosing
	Able and willing to be contacted via telephone for all necessary telephone contacts
	Agree to use an effective form of contraception if of child-bearing potential for the duration of medication dosing
	Must provide a contact/support person if they are unreachable by study staff or in the event of severe distress or suicidality
	Agree to not use any medications on the prohibited medications list during the study
	Agree not to take any herbal supplement for the duration of medication dosing (except with prior approval of the research team)
Demographics	At least 25 years old
	Proficient in speaking and reading English
Clinical characteristics	Diagnosis with an incurable stage IV incurable solid organ malignancy
	Prognosis of at least 6 months life expectancy from the time of screening
	Moderate distress (4 or greater) as measured by the distress thermometer

**Table 2:** Full exclusion criteria

	Exclusion criteria
Treatment	Currently participating in a clinical trial of a systemic anti-cancer treatment
Physical health	Pregnant or lactating BMI < 18.5
	Diagnosis of cerebral metastases
	Karnofsky performance scores below 50 or other physical limitations that preclude participation in weekly psychotherapy and microdosing of LSD
Lab work	Liver function test > 3 times the upper limit of normal or creatinine clearance < 30 mL/min
Diagnosis	Have a current diagnosis or history of any medical condition that could make receiving a sympathomimetic drug harmful because of potential increases in blood pressure and heart rate as assessed by a study physician
Vital signs	Cardiovascular conditions including abnormal heart rate seen by ECG
	Blood pressure not exceeding 160 mmHg (systolic) and 90 mmHg (diastolic) (measured at three time points)
Mental health	
Diagnosis	Lifetime history of schizophrenia or other psychotic disorders, or bipolar I or II disorder as assessed by the Standard MINI (Standard version 7.0.2)
	A current diagnosis of PTSD, panic disorder, agoraphobia, OCD, anorexia, and bulimia as assessed by the Standard MINI (Standard version 7.0.2)
Current risk	Elevated risk of suicide as determined by the Columbia-Suicide Severity Rating Scale (C-SSRS) or by a study psychiatrist
Drug use	Any lifetime history of psychedelic microdosing; defined as repeated low-dose psychedelic usage for more than a week at a time
	Use of a psychedelic within the last year
	Recent or current use of illicit drugs including methamphetamine, heroin, and synthetic cannabis. Other non-prescribed drugs will prompt exclusion at the discretion of a study physician
	Current THC/cannabis usage will prompt exclusion if the participant does not agree to cease. However, CBD is permitted, and usage will be recorded

Before attending the screening visit, potential participants and their nominated family member will be provided with a participant information sheet and consent form detailing the nature of the trial (including education on LSD microdosing and MCP), the implications and constraints of the protocol, the known side effects, and any risks involved in taking part. Participants will have time to consider the information and the opportunity to question the investigator, their usual care provider, or other independent parties to decide whether to participate. Written informed consent will then be obtained by means of the participant’s dated signature and the dated signature of the person who presented and obtained the informed consent. The person who obtains consent will be an investigator in the trial. A copy of the signed informed consent form will be given to the participant. Continued eligibility and verbal consent will be reconfirmed at the start of each visit to the study site. At the screening visit, following consent processes, participants will be checked for eligibility (including recording and reviewing all medication and cancer-related treatment) and, if eligible, are approved for inclusion by study staff.

After blood test results have been received, participants will be contacted to inform them of their continued eligibility and to book their first treatment session. On arrival at the first treatment session (or day prior), participants will complete several baseline psychometrics and physical health checks (vital signs, serotonin syndrome checks). Participants will report any changes in health status or medications at each visit and in each dose day questionnaire. Participants will then receive a single dose of the drug they have been randomised to receive (see Drug Preparation and Administration below). Vital signs, serotonin syndrome checks, and subjective drug effects VAS will be completed at 30, 120, 240, and 360 min following dosing. At 45–60-min post-dosing, the participant will attend their first 1-h MCP session. Participants will be discharged with four additional home doses intended to be taken on days 4, 11, 18, and 25 (± 1 day).

All participants will receive 7 sessions of MCP on days 1, 8, 15, 22, 29, 36, and 43 (± 1 day), notwithstanding the potential need for break weeks (see below). The MCP sessions (refer to Table 3) will be conducted at either the research clinic site or via Zoom by a registered psychologist who has been trained and has expertise in delivering MCP. Remote delivery of MCP via Zoom may be necessary in certain situations such as where participants cannot attend due to illness or have medical appointments. The home supply method has been designed to account for any necessary switches from in-clinic to remote delivery of MCP so that participants will always have a dose available to take before their MCP session.

**Table 3:** Weekly topics and goals of MCP sessions [21]

Session	MCP Weekly topics
1	**Concepts and Sources of Meaning: Introduction and Overview**
	Session goals: Learn patient’s cancer story and introduce concepts and sources of meaning
2	**Cancer and Meaning: Identity Before and After Cancer Diagnosis**
	Session goals: Develop a general understanding of one’s sense of identity and the impact cancer has made upon it
3	**Historical Sources of Meaning: Life as a Living Legacy (past, present, future)**
	Session goals: Develop an understanding of one’s legacy through exploration of three temporal legacy modes; the legacy that has been given from the past, the legacy that one lives in the present, and finally, the legacy one will leave in the future. Participants also begin developing a Legacy Project
4	**Attitudinal Sources of Meaning: Encountering Life’s Limitations**
	Session goals: Explore one of Frankl’s core therapeutic principals; ultimately, we have the freedom and capacity to choose our attitude toward suffering and life’s limitations and to derive meaning from that choice
5	**Creative Sources of Meaning: Engaging in Life via Creativity and Responsibility**
	Session goals: Develop an understanding of the significance of ‘creativity’ and ‘responsibility’ as important sources of meaning in life
6	**Experiential Sources of Meaning: Connecting with Life via Love, Nature, and Humour**
	Session goals: Foster an understanding of the significance of connecting with life through experiential sources of meaning—particularly through experiencing love, beauty, and humour
7	**Transitions: Reflections and Hopes for the Future**
	Session goals: Review the sources of meaning. Review of the Legacy Project. Reflections on the lessons and impact of the therapy, discussion of hopes for the future, and the transition from being in the therapy to enacting the lessons learned in daily life as the therapy comes to an end

The schedule and process of dosing are tied to the MCP treatment. Dosing will occur in the clinic when participants attend each MCP session, i.e. days 1, 8, 15, 22, 29, 35, and 43 (± 1 day). On these days, doses will be supplied on-site by study staff, so participants do not need to remember to take their medication with them into the clinic. On dosing days between MCP sessions, i.e. days 4, 11, 18, 25, 32, and 39 (± 1 day), doses will be self-administered at home. This dosing pattern will be repeated for a total of 13 occasions over a 43-day period on days 1, 4, 8, 11, 15, 18, 22, 25, 29, 32, 35, 39, and 43 (± 1 day). All doses will be administered by 2 pm at the latest to minimise potential disruptions to sleep. At MCP session 4, participants will be resupplied with the final two home doses (days 32 and 39 (± 1 day)). If participants are unable to attend the clinic on this day, a member of the study team will conduct a home delivery of their final medication doses.

A key feature of the research design is the inclusion of potential break weeks. Given the health status of this population, breaks in therapy may be necessary if a participant is unwell due to their cancer or has treatment or other medical commitments. In these situations, participants will take a 1-week break from MCP and medication dosing and resume participating in the protocol the following week. Seven break weeks are available to each participant. If all seven break-weeks are utilised, the treatment period would be extended to a maximum of 13 weeks and 1 day.

Participants have the right to withdraw from the trial at any time. In addition, the investigator may discontinue a participant from the trial at any time if the Investigator considers it necessary for any reason including patient death, eligibility violations, significant non-compliance with treatment or trial requirements, an adverse event or medical condition requiring discontinuation, withdrawal of consent, or loss to follow-up. Significant non-compliance is where a participant falls below feasibility criteria (see Table 4) without having a justifiable health or cancer treatment-related reason.

**Table 4:** Primary measures

Outcome domain	Measure	Definition
Feasibility	Adherence to medication regimen	Compliance with study treatment (at minimum 80% of doses taken indicates feasibility, i.e. 11 out of 13 doses taken)
	Attendance at MCP session	Percentage of participants attending 4 out of 7 sessions
	MCP treatment fidelity	Rating on the Memorial Sloan Kettering Fidelity Rating Scale. A 5-item yes/no indicating presence of content, a 5-item 3-point Likert scale measuring coverage, and a final 5-point Likert scale assessing overall focus on meaning/purpose
	Participant recruitment	Percentage of consented participants randomised (70% or greater indicates feasibility)
	Attrition	Number of dropouts following randomisation (30% or less indicates feasibility)
Acceptability	Qualitative interviews	Semi-structured qualitative interviews at baseline and at 1 month following treatment completion (T8) (participant and support person) and will address expectations and the acceptability of study procedures
	Completeness of data	Percentage of complete data
Safety	Vital signs	Monitoring of vital signs including heart rate, blood pressure, and body temp at 0, 30, 120, 240, and 360 min post-administration at T1. Also measured at 0- and 45-min post-administration, for T2–T7. Serotonin syndrome checks on every in-clinic day T1–T7
	Palliative Care Outcomes Collaborative Symptom Assessment Scale (PCOC-SAS)	Nine symptom items on an 11-point scale of distress, rated from ‘absent’ to ‘severe’
	Adverse events	Participants are asked on dosing days to report any ‘unpleasant health effects’ and to rate them as mild, moderate, or severe
	ECG data	ECG data will be reviewed by a study physician, noting where reference ranges are exceeded

### Outcomes

Primary and secondary outcome measures are outlined in Tables 4 and 5. Feasibility measures include adherence to medication regimen, attendance at MCP sessions, MCP treatment fidelity, and participant recruitment and attrition rates; the feasibility targets (detailed below) are based on previous research [21, 22]. Acceptability will be assessed using open-ended questions (T0–T9) and semi-structured qualitative interviews at baseline and at the one and 6-month follow-ups. Furthermore, the completeness of data will also be used to indicate the acceptability of completing study measures. Safety assessments include physiological data (HR, BP, body temperature), weekly symptom reporting, adverse event reporting, and ECG taken on the last study visit.

**Table 5:** Secondary measures

Outcome domain	Measure	Scale
Sense of meaning	Personal Meaning Index of the Life Attitude Profile – Revised (LAP-R) [56]	16 items, 7-point Likert scale from 1 (strongly agree) to 7 (strongly disagree). Scores are summed
Quality of life	Functional Assessment of Chronic Illness Therapy – General (FACT-G) [57]	27 items, 5-point Likert scale from 0 (not at all) to 4 (extremely). Four subscales, summed
Spiritual well-being	Functional Assessment of Chronic Illness Therapy – Spiritual Well-being 12-item scale (FACIT-SP-12) [58]	12 items, 5-point Likert scale from 0 (not at all) to 4 (very much). Scores are summed
Sense of connectedness	Watts Connectedness Scale [59]	19 items, rated on VAS (0–100), anchors: ‘Not at all’ and ‘Entirely’. Three subscales, reported as mean scores and subscale means
Anxiety and depression	Depression, Anxiety and Stress Scale (DASS-21) [60]	21 items, 5-point Likert scale, from 0 (never) to 4 (almost always). Three subscales, reported as summed scores (each subscale sum multiplied by two)
	Hospital Anxiety and Depression Scale (HADS) [61]	14 items, 5-point Likert scale (0–4). Two subscales, reported as summed scores
Te Whare Tapa Wha Mental Health outcomes	Hua Oranga [62]	14 items, 5-point Likert Scale (1–5). Four subscales, reported as subscale or total questionnaire summed scores
Demoralisation	Demoralization Scale [63]	24 items, 5-point Likert scale, ranging from 0 (never) to 4 (all the time). Five subscales, reported as summed scores
Attitudes towards death	Schedule of Attitudes towards Hastened Death (SAHD) [64]	20 items, scored 0 (false) or 1 (true), reported as a total sum
Personality	Big Five Inventory (BFI-2-XS) [65]	15 items, 5-point Likert scale, ranging from 1 (disagree strongly) to 5 (agree strongly). Five subscales, reported as summed scores
Therapeutic alliance	Working Alliance Inventory Short Form (WAI-SF) [66]	12 items, 5-point Likert scale, ranging from 1 (seldom) to 5 (always). Three subscales, reported as summed scores
Altered states of consciousness	5-Dimensional Altered States of Consciousness Rating Scale (5D-ASC) [67]	94 items, with 5 scales and 11 subscales reported as % of the maximum score
Personality: absorption	Modified Tellegen Absorption Scale (MODTAS) [68]	34 items rated on a 5-point scale (0–4) reported as the sum of scores (0–136)
Treatment expectancy	Treatment Expectancy Questionnaire [69]	15 items rated on an 11-point Likert scale
Pain	Brief Pain Inventory – Short Form (BPI-SF) [70]	4 severity items and 7 interference items, rated on an 11-point Likert scale, ranging from 0 (no pain/does not interfere) to 10 (pain as bad as you can imagine/completely interferes), the two subscales are reported as means
Measurement of blinding	Dose Day Questionnaire	Single-item measure (placebo, LSD, don’t know)
Subjective drug effects	Drug Effects Visual Analogue Scale [39]	12 items, rated on VAS (0–100), reported individually and as mean score
Caregiver stress – support person only	Kingston Caregiver Stress Scale [71]	10 items, rated on 5-point Likert scale, reported as the sum of scores (10–50)
Closeness to others – support person only	Inclusion of Other in Self [72]	Single-item measure, between 1 and 7

The schedule of assessments is detailed in Fig. 1. Key time points for psychometrics are days 1, 22, and 43, as well as the 1- and 6-month follow-ups (see Fig. 1). In addition to psychometrics, vital signs and serotonin syndrome checks will be conducted at every in-clinic visit. Participants will also record adherence to dosing, subjective drug effects, measurement of blinding, and any adverse events on home dosing days.

### Participant recruitment

Adult Māori (*n* = 20) and non-Māori (*n* = 20) patients with a diagnosis of stage IV cancer and depression and/or anxiety will be referred by regional cancer services or patient self-referral. Participants must be willing for the Investigators to communicate directly with their medical team to determine suitability and whether they have adequate physical status for study participation. Recruitment is estimated at one participant per week for 40 weeks (10 months) based on the recruitment rate of cancer patients through a similar study [73]. Interested participants will contact researchers to express interest after seeing advertisements or will provide consent to be contacted by researchers following referral by health professionals.

### Randomisation, masking, and code-breaking

A biostatistician (Author AC) will perform randomisation of the allocation of participants to the interventions. The biostatistician will generate a randomisation code list, and participants will be randomised in randomly permuted blocks with stratification used to give separate lists for Māori and non-Māori. The active and placebo interventions will be matched in appearance. Only the biostatistician and pharmacists involved in the study will be unblinded to study group allocation. These persons will not interact with study participants and will not be present during any drug administration sessions. To ensure allocation concealment when a participant enters the trial, they will then be allocated by a blinded investigator to the first available code on the randomisation sequence list. Both participants and Investigators will be kept blinded until the End of Trial. The Start of Trial is the date of the first screening visit for the first potential participant. The End of Trial is the date of the last 1-month follow-up interview of the last participant. Each participant is considered an active trial participant from the first screening visit until 1-month follow-up unless otherwise withdrawn. In case of emergency, study pharmacists will also keep an electronic spreadsheet of allocations so that de-blinding can be performed rapidly.

### Drug preparation and administration

Good Manufacturing Practice (GMP) quality LSD Hemitartrate Active Pharmaceutical Ingredient (Psygen Ltd, Calgary, Canada) will be formulated to GMP by Biocell Corp (Auckland, New Zealand) to produce MB-22001—the investigational medicinal product (IMP) to be used in this trial. The Contract Manufacturer will receive a MedSafe Manufacturing license for the IMP prior to manufacturing the first clinical trial batch. Investigational products will be labelled consistent with legal requirements. All participants will be offered a lockbox to securely keep the IMP in at home—and to prevent accidental ingestion by minors. MB-22001 is a liquid formulation that participants can self-administer sublingually.

### Titration protocol

To ensure participant acceptability of dosage, this study will utilise a titration protocol. LSD is known to have stimulant-like effects, even at low doses, which can result in feelings of uneasiness or overstimulation [39]. This effect can vary from person to person. Flexibility in dosage ensures participants will be receiving an appropriate dose, reducing the likelihood of negative side effects that would be counter-productive to the purpose of the trial. Based on findings from the MDLSD study, the starting dose will be 8 µg, increasing or decreasing at a rate of 1 or 2 μg per dose. This is lower than the dose in the MDLSD study (10 µg). The maximum dose given to participants will be 20 μg. The decision to increase the dose will be based on participant feedback and psychometrics, such as the subjective drug effects questionnaire. The most appropriate dose for a participant will be one in which they may feel subtle effects of the LSD, but not to the extent that effects are negative, overstimulating, or consciousness altering. Participants will be informed that if they experience any disturbance of daily functioning they should decrease the dose for the next dosing.

Participants will use a 5-point Likert scale in the dose day questionnaires to rate the tolerability of the previous dose. Doses will then be decreased in 2 or 1 μg increments if the last dose had any tolerability issues (i.e. was rated as ‘too much’) or increased by 2 or 1 μg increments if they feel no effects at all and rated the dose as inadequate.

To reduce the likelihood that titration procedures will impact blinding, participants will be informed that it is not uncommon for people to increase their dosage to 20 μg and not feel anything. Titration procedures will be aided by an in-app questionnaire, asking for subjective experience of their last dose, with oversight from the study team.

### Relevant concomitant care and post-trial care

Participants will continue to receive standard care from their GP or wider medical team for the duration of the trial. Long-term harm to participants is considered highly unlikely; however, participants can apply for compensation for any injury sustained during the trial under the Auckland University insurance policy.

### Sample size

Given the primary aim of this study to establish the feasibility, acceptability, and safety of PA-MCP for future investigation in a fully powered clinical trial, this study is not powered or intended to determine statistical significance [74]. With the proposed sample of 40 participants, we are able to estimate a compliance rate of 80% to within a 95% CI of within ± 12%. This sample size will provide sufficient data to assess feasibility, acceptability, and safety metrics based on a recent study investigating psychedelic-assisted psychotherapy in cancer patients (*N* = 29; Ross et al., 2016) and previous MCP studies where 66–71% of participants completed all 7 psychotherapy sessions [21, 22].

### Statistical analyses

Baseline measures will be presented for each treatment group using summary statistics, with frequencies and percentages for categorical variables and means with appropriate measure of spread for continuous variables. Data on quantitative primary outcome measures for feasibility, acceptability, and safety for Māori and non-Māori participants including recruitment rates, attrition, adherence to medication and MCP, adverse effects, MCP treatment fidelity, and feasibility of outcome data collection will also be presented using descriptive statistics.

Changes in secondary outcome measures over the study period will be assessed using generalised linear mixed models (GLMMs) with main effects of treatment (PA-MCP versus Placebo-MCP) and time (T0–T9), treatment-by-time interactions, and subject-level random effects to model longitudinal trajectories whilst accounting for correlations between repeated measures within subjects.

Qualitative interviews will be analysed using thematic analysis, a systematic process for identifying patterns in qualitative data [75]. Interviews will be transcribed and read in detail to gain familiarity with the data. The transcripts will be coded to identify emergent features; these codes will then be reviewed, refined, and organised to produce themes. Though this process will be reflexive and iterative, themes will be considered in relation to our primary outcomes, as well as those relating to experience, expectations/knowledge, and psychological wellbeing. Finally, these themes will be considered in context of broader trial findings and the wider literature.

### Adverse event reporting and harms

An adverse event (AE) is any untoward medical occurrence (e.g. any unfavourable and unintended sign [including abnormal laboratory findings], symptom, or disease) in a participant after being confirmed into the trial until the night of the last study visit. Therefore, an AE may or may not be temporally or causally associated with the use of the investigational medicinal product. Any intentional misuse and abuse of the product and consequences thereof are also considered an adverse event irrespective if a clinical event has occurred.

AEs include any medical occurrence in a participant, including any abnormal sign (e.g. abnormal physical exam or laboratory finding), symptom, or disease, temporally associated with the participant’s involvement in the research, whether or not considered related to participation in the research. This definition includes concurrent illnesses or injuries and exacerbation of pre-existing conditions. They do not include anticipated day-to-day fluctuations of pre-existing disease(s) or condition(s) present or detected at the start of the study that do not worsen.

All AEs occurring on-site will be recorded by study staff in the CRF, whether or not attributed to trial medication. Any adverse effects when participants are off-site on dose days only will be recorded via participants’ daily report form. AEs recorded in the daily report form will be reviewed daily.

Events judged to be Serious Adverse Events will be reported to MedSafe as per Sect. 6 of the MedSafe reporting guidelines described in the Regulation of Therapeutic Products in New Zealand Part 11: Clinical trials – regulatory approval and good clinical practice requirements. These are submitted via the HDEC website (https://nz.forms.ethicalreviewmanager.com) as soon as is practical. In addition, SAEs will be reported in real-time to the HRC Data Monitoring Committee.

### Data and safety monitoring

The trial will be overseen by a Trial Steering Committee (TSC). The TSC will be comprised of a subset of the Investigators of this study. The roles of the TSC are to collaboratively develop and approve the final protocol; oversee trial progress, check adherence to the protocol, assess participant safety, and consider new information; and be responsible for publication and dissemination. The TSC was in full agreement prior to the submission of the final protocol. The TSC will take responsibility for major decisions such as a need to change the protocol for any reason, monitoring and supervising the progress of the trial, and reviewing relevant information from other sources, where at least 50% of the Investigators including the PI must be in agreement. The PI has a deciding vote.

Data monitoring for this trial will be conducted by an independent Data Management Committee (DMC) run by the Health Research Council of New Zealand (HRC). Data review meetings will be conducted every 6 months from the commencement of the trial until its termination. Open and closed reports will be prepared by the trial statistician for each meeting using data current to within two months of the meeting and will be submitted to the HRC 14 days before DMC meetings. The data review meetings will consist of two sessions: a closed session attended only by the statistician who prepared the DMC reports and an open session where the principal investigator will provide the DMC with an update on the trial and any other relevant information and answer questions that were raised by the reports. Protocol amendments will be submitted to the DMC, as well as Medsafe and the approving Ethics Committee.

### Data collection and management

Case report forms (CRFs) will be entered into the online Research Electronic Data Capture (REDCap) tool hosted at the University of Auckland by study researchers for each participant. This will include demographics, medical history, height, weight, current medications, notes on physical examinations, vital signs, and adverse events at the study site. REDCap is a secure, web-based software platform that supports data capture for clinical trials.

ECG results will be electronically appended to the CRF. All subsequent psychometric measures will be completed by participants directly into REDCap, and qualitative interviews will also be captured electronically. Serum chemistry and haematology, biomarker, and pharmacokinetic data will be received electronically from subcontracted laboratories. Tissue samples will be used for analyses as described in the protocol for screening purposes only. No tissue samples will be kept for the purposes of the study or for further analysis.

All electronic data will be stored on secure University of Auckland servers, which include password protection, multi-site backups, and tape archiving. An original, unprocessed version of every data file will be kept on the servers such that these files may only be modified by a University of Auckland IT systems administrator, thus ensuring the fidelity and audit capability of all electronic data. Scanned versions of all paper-based CRFs and source data formats will be made and held on the servers in password-protected files to ensure the fidelity of these data and allow future audits of extracted data.

Participants will be identified by a unique trial-specific number and/or code in any electronic database. On all trial-specific documents, other than the signed consent form, prescriptions, and page one of the CRF (separately filed), the participant will be referred to by the trial participant code, not by name. All source data including that contained in the CRFs and the Trial Master File (TMF) will be held for a period of 15 years from the completion of the trial.

De-identified and/or anonymised data will be stored on university-managed storage systems and may be shared with both international and national collaborators/companies/researchers on request for future research or added to data from other sources to form larger datasets. Participant sessions will be video recorded and stored in password-encrypted files on university-managed storage, and university credentials will be required for data access. Access will be restricted to named people on the study, or those persons who sign a confidentiality agreement with the University of Auckland. Video recordings are essential to enable the assessment of treatment fidelity by a suitably trained member of the study team. Thirty percent of the total participant sessions will be watched and rated for treatment fidelity. Training for the fidelity assessor will be conducted by an international expert in MCP.

### Dissemination policy

Results will be published in relevant academic journals and communicated with the wider public via news media and social media. Participants can submit a request to view their own data at any time.

## Discussion

Timely and effective care is essential at end-of-life to reduce the significant distress that often arises with a life-limiting illness. This study will provide the first opportunity to test the feasibility, acceptability, and safety of LSD microdosing alongside MCP for advanced-stage cancer patients compared to MCP alone. This placebo-controlled, double-blind, randomised feasibility trial will pave the way for a larger scale RCT powered to determine efficacy. To date, no research has been conducted on psychedelic microdosing-assisted psychotherapy in cancer patients. This treatment paradigm offers potential benefits to patients in that it may expedite or increase the efficacy of standard psychological treatment and provide an alternative to full-dose psychedelic therapy. Microdosing has the potential to be less psychologically intensive and burdensome compared to high-dose psychedelic experiences.

This study’s aims are to assess the feasibility, acceptability, and safety of psychedelic microdosing-assisted meaning-centred psychotherapy. The delivery of microdoses alongside psychotherapy in cancer patients has not yet been researched; cancer patients are vulnerable with complex health and psychological needs, and these characteristics make conducting research and designing new interventions more challenging. This feasibility trial is critical in establishing an appropriate treatment regimen that is not unduly burdensome. These findings will form the foundation of a larger-scale RCT to test the efficacy of this potential treatment. There are several design factors to consider when working with an unwell population who may be receiving concurrent cancer treatment including the appropriate dosage and dosing schedule, the practicality of attending weekly MCP sessions, and the acceptability of MCP delivered over Zoom as an alternative to in-clinic sessions. This feasibility trial will also give an indication of recruitment patterns and levels of interest in the study population. Although previous research has indicated that there would likely be a high degree of uptake of psychedelic-assisted psychotherapy in this sample [76], this is the first study of its kind to offer this treatment. Furthermore, attrition rates will not only inform future RCT design but will provide valuable data on feasibility and acceptability. Finally, collecting rigorous safety data is essential for ongoing research and the potential future implementation of psychedelic-assisted therapy into general health practices.

At the feasibility stage of research, incorporating qualitative methodologies is important. In this current work, qualitative interviews conducted at baseline and at the 1- and 6-month follow-ups will provide rich data that will complement and expand on quantitative findings. Semi-structured interviews will ask participants about their expectations, knowledge of psychedelics, their experience of taking medication, attending MCP sessions, completing the study measures, and about any benefits or negative effects of the intervention. Family members are also included in the study design by asking them to complete brief psychometrics and qualitative interviews at baseline and 1-month follow-up. This data will add richness and build on data collected from participants which will benefit future work.

Compared to many other randomised controlled trials assessing psychedelic therapies, this trial has aimed to better reflect the real-world settings in which such treatments may eventually take place. Decisions about the inclusion and exclusion criteria, as well as the dosing protocol, reflect this realism. These key decisions include allowing participants to be currently undergoing noncurative treatment and allowing antidepressants and pain medication as concomitant medications. It is possible that future trial participants or those who seek out psychedelic-assisted therapy as treatment may be undergoing noncurative cancer treatment to improve quality of life or prolong survival. Including these patients from the early stages of feasibility will provide a more accurate assessment of potential challenges or benefits, ensuring that future interventions are designed with these patient needs in mind.

Similarly, patients may be taking antidepressants to help manage anxiety or depression. Although research to date [77] and theoretical work [78] suggests a good degree of safety, some of this literature is observational or followed historic scientific standards. It is therefore imperative for a well-designed and rigorous scientific study to confirm these findings. Finally, this protocol integrates both supervised (in-clinic) dosing and unsupervised (at-home) dosing, allowing for a more naturalistic assessment of microdosing and its potential benefits. Excluding the recent MDLSD study completed at the University of Auckland [39], practically all RCTs to date of microdosing have been conducted in laboratories [38, 43–46] which produces limited ecological validity and may influence the resultant findings due to the widely acknowledged importance of set and setting. In summary, this protocol outlines a novel exploration of the feasibility, acceptability, and safety of psychedelic microdosing-assisted meaning-centred therapy that will add to the growing scientific literature and help to build our understanding of the therapeutic benefits of psychedelics.

### Trial status

The PAM trial protocol is currently on version 1.6. Recruitment for this trial commenced in September 2023 (*n* = 2) and will run through to the anticipated completion of the trial in late 2024.

### Trial sponsor and role of sponsor

The study sponsor is the University of Auckland, contactable via the Office of Research Strategy and Integrity at humanethics@auckland.ac.nz. The study sponsor has no involvement in the study design; collection, management, analysis, and interpretation of the data; writing of the report; and the decision to submit the report for publication.

### Supplementary Information

**Additional file 1.**

**Additional file 2.**

## Data Availability

The corresponding author will release documentation including PIS, consent forms, and study advertisements on publication of trial results. Access to the final trial dataset will only be available to the study investigators, DMC, and any other relevant regulatory bodies.

## Abbreviations

5D-ASC: 5-Dimensional Altered States of Consciousness
ADHB: Auckland District Health Board
AE: Adverse event
AKPS: Australian Karnofsky Performance Score
ANZCTR: Australia New Zealand Clinical Trials Registry
BFI-2-XS: The Big Five Inventory-2 Extra Short Form
BMI: Body mass index
CBD: Cannabidiol
CRF: Case report form
CSSR-S: Columbia-Suicide Severity Rating Scale
DASS: Depression, Anxiety, and Stress Scale
DMC: Data Monitoring Committee
ECG: Electrocardiogram
FACIT-SP-12: Functional Assessment of Chronic Illness Therapy–Spiritual Well-being 12
FACT-G: Functional Assessment of Cancer Treatment–General
GMP: Good manufacturing practice
GP: General practitioner
HDEC: Health and Disability Ethics Committee
HRC: Health Research Committee
IMCP: Individual meaning–centred psychotherapy
IMP: Investigational medicinal product
LAP-R: Life attitude profile revised
LSD: Lysergic acid diethylamide
MAOI: Monoamine oxide inhibitor
MCP: Meaning-centred psychotherapy
MDLSD: Microdosing LSD study
Mini-ACE: Mini-Addenbrooke’s Cognitive Examination
MODTAS: The Modified Tellegen Absorption Scale
OCD: Obsessive compulsive disorder
PA-MCP: Psychedelic assisted meaning–centred psychotherapy
PCOC-SAS: Palliative Care Outcomes Collaboration Symptom Assessment Scale
PI: Principal investigator
PIS: Participant information sheet
PTSD: Post-traumatic stress disorder
QOL: Quality of life
SAE: Serious adverse event
SAHD: Schedule of attitudes towards hastened death
SCOTT: Standing Committee on Therapeutic Trials
SNRI: Serotonin and norepinephrine reuptake inhibitor
SOP: Standard operating procedure
SSRI: Selective serotonin reuptake inhibitor
TEX-Q: Treatment Expectancy Questionnaire
THC: Tetrahydrocannabinol
TMF: Trial master file
TSC: Trial Steering Committee
VAS: Visual Analogue Scale
WAI-SF: Working Alliance Inventory–Short Form
WCS: Watts Connectedness Scale
