# Supplementary material for: PAM trial protocol: a randomised feasibility study of psychedelic microdosing–assisted meaning-centred psychotherapy in advanced stage cancer patients
Source: Pilot Feasibility Stud. 2024 Feb 12;10:29. doi: 10.1186/s40814-024-01449-9 (PMC10860284; doi:10.1186/s40814-024-01449-9)
Supplement: Supplementary file 1 — Additional file 1. [file 40814_2024_1449_MOESM1_ESM.pdf]

Participant Information Sheet

Feasibility study of Psychedelic Microdosing-Assisted Meaning Centred Psychotherapy in advanced stage cancer patients

Sponsor: The University of Auckland

Auckland 1023, New Zealand
Lead Researcher: Dr Lisa Reynolds

Study Site: The University of Auckland

Contact phone number: 021 064 8489

Ethics committee ref.: 2022 FULL 13074

Nāu te rourou, nāku te rourou, ka ora ai te iwi
With your food basket and my food basket the people will thrive

You are invited to take part in a study on the effects of combining a talk therapy called meaning-centred psychotherapy with repeated microdoses (very small doses) of LSD on psychological well-being and cancer-related distress. Please note, this is not intended to treat your cancer, the intervention is designed to target psychological symptoms only. Whether or not you take part is your choice. If you don't want to take part, you don't have to give a reason, and it won't affect the care you receive. If you do want to take part now, but change your mind later, you can pull out of the study at any time.

This Participant Information Sheet will help you decide if you'd like to take part. It sets out why we are doing the study, what your participation would involve, what the benefits and risks to you might be, and what would happen after the study ends. We will go through this information with you and answer any questions you may have. You do not have to decide today whether or not you will participate in this study. Before you decide you may want to talk about the study with other people, such as family, whānau, friends, or healthcare providers. Feel free to do this.

If you agree to take part in this study, you will be asked to sign the Consent Form on the last page of this document. You will be given a copy of both the Participant Information Sheet and the Consent Form to keep.

This document is 16 pages long, including the Consent Form. Please make sure you have read and understood all the pages.

VOLUNTARY PARTICIPATION AND WITHDRAWAL FROM THIS STUDY

Your participation in this study is entirely voluntary. It is up to you if you take part or not. If you do decide to take part you will be given this information sheet to keep and be asked to sign a consent form. If you don't want to take part you don't have to give a reason. If you decide to take part you are still free to withdraw at any time and without giving a reason. A decision to withdraw at any time, or a decision not to take part, will not affect the standard of care you receive from us. However, if you do withdraw from the study prematurely, for your benefit we will ask you to remain at our research facility until it is safe for you to leave.

If you take part in the study you have the right to access any information about you collected during the study after your completion or withdrawal from the study.

If we learn anything about your health status or the medications to be tested during the study during the study that affects your health you will be informed of this.

Any information you give us about yourself will remain private and confidential.

What is the purpose of the study?

- The goal of our research is to test whether taking very small (micro) doses of LSD has effects on psychological wellbeing when combined with a talk therapy called meaning-centred psychotherapy. Your participation will help us test these claims.
- We are investigating whether microdosing of LSD affects how meaning-centred psychotherapy works for people with cancer. Other studies have shown that LSD in large doses has psychedelic properties which include temporary alterations or distortions in perception. In large doses, LSD has been shown to reduce psychological distress in cancer patients. Our study involves very small doses of LSD and does not have psychedelic effects. Many users claim that taking very small doses of LSD can have positive effects on personality, creativity and mood. However, this has not been tested in a formal study with cancer patients and these claimed effects may or may not be real. It has also been claimed that microdosing with LSD might work well in conjunction with talk therapy to improve wellbeing.
- This study is a placebo-controlled trial, meaning that half of the doses administered will be LSD and half will be a placebo (either inactive (saline) or active (caffeine or ritalin)). These placebos have been chosen to mimic the effects of LSD microdoses so that any results we find are not due to our or your expectations regarding the drug. The study is "double-blinded" so neither you or we will know what you have been given until the end of the trial which might take several years to complete.
- We are a group of scientists and clinicians based at the University of Auckland and the Auckland District Health Board who are studying psychedelic assisted psychotherapy. This study is being done as part of a student's PhD thesis. This study has been approved by a Health and Disability Ethics Committee. Contact details are given at the end of this sheet.
- This study is funded by the Health Research Council of New Zealand and MindBio Therapeutics Ltd.

How is the study designed?

We are aiming to recruit 40 participants across Auckland. This study will take up to 25 hours of your time and involve eight visits to our research centre. This includes a screening session and seven sessions of talk therapy. There are some situations (e.g., if you feel too unwell to travel or if you are isolating due to Covid-19) when sessions 2-7 would be

completed remotely via teleconference (e.g., using Zoom). We will discuss this possibility with you during the first session and make sure you are comfortable with this.

All participants will receive a talk therapy called meaning-centred psychotherapy. In addition, participants will be randomised to either receive LSD microdoses or a placebo alongside their psychotherapy. In the placebo condition, participants will receive one of three types of placebos. These include an inactive placebo or one of two active placebos, either caffeine or Ritalin. Across the treatment period, participants will receive a total of 13 doses of either LSD or one of the placebos. Finally, there will be a follow-up interview one month and six months after the end of treatment period. The LSD doses in this trial start at 8 micrograms and go up to 20 micrograms. This is between about 1/10 and 1/5 of the typical dose of LSD used when people take LSD recreationally.

As a part of this study the following tests will be undertaken:

- A simple blood test will be conducted during the first screening visit. This blood test will look at simple health markers (e.g. liver function) so that we can confirm it is safe for you to participate in this research. No further blood testing will be required across the duration of the study. These samples will be sent to LabPlus to be analysed and disposed of. As the blood samples are unable to be returned from LabPlus, karakia at the time of destruction will not be available.
- We will conduct an Electrocardiogram (ECG) during the screening session and at the final treatment session. This is a simple and short procedure that measures the electrical activity generated by your heart. To do this, leads will be placed on your chest. Just like the blood test, the results from this ECG will provide information about your heart health and allow us to confirm that your participation is safe.
- We will also monitor your blood pressure and heart rate during on-site study sessions.
- We will also ask you to complete some psychological questionnaires on your dosing days.

WHO CAN TAKE PART IN THE STUDY?

You have been chosen to take part in this study because you have an advanced cancer diagnosis and experience moderate to severe anxiety or depression. You must be at least 25 years old and be proficient in speaking and reading English. Our study medical team will help determine if you meet the criteria for inclusion in this study.

You will not be eligible to participate in this study if you:

- Do not have a stage IV incurable solid organ malignancy.
- Are already participating in a clinical trial of anti-cancer treatment.
- Are pregnant or lactating.
- Have a Body Mass Index (BMI) lower than 18.5.
- Experience severe limitations in physical mobility, to the extent that you may not feel well enough to attend weekly in-clinic sessions.
- Have a current or past diagnosis of schizophrenia or other psychotic disorders, or bipolar I or II disorder. Or a current diagnosis of PTSD, panic disorder, agoraphobia, OCD, anorexia, or bulimia.
- Have, or have had, a certain medical condition other than the cancer diagnosis. For example: kidney, liver, or heart conditions.

- Are feeling suicidal.
- Have previous experience microdosing psychedelics for more than a week at a time.
- Have used psychedelics in the past year.
- Recent or current use of illicit drugs including methamphetamine, heroin and synthetic cannabis.
- Current use of THC/cannabis.

WHAT WILL MY PARTICIPATION IN THE STUDY INVOLVE?

As mentioned above, this study will take up to 25 hours of your time and involve eight visits to our research centre. We describe these visits below and have added a table at the bottom of this section to help you understand the timing of the visits and what happens at each visit.

You are welcome to bring a family/whānau member or other support person to any clinic visit

Initial screening visit: On the first day, you will come to our centre and we will confirm your eligibility to take part in the study. This session will take approximately 2 hours. We will ask you questions about your cancer diagnosis and treatment as well as other aspects of your physical and mental health. We will also need to ask about your drug use history to confirm whether you are eligible to take part. We will take blood samples to test your physical health (liver and kidney function). A physical health assessment will be performed including an ECG recording of your heart.

You are encouraged to consult with your whānau, hapū or iwi regarding participation in this project. You may hold beliefs about a sacred and shared value of all or any tissue samples removed. The cultural issues associated with sending or storing your tissue should be discussed with your family/whānau as appropriate. There are a range of views held by Māori around these issues, some iwi disagree with storage of samples citing whakapapa and advise their people to consult prior to participation in research where this occurs. We acknowledge that you have the right to choose.

If you are eligible and decide to take part, we will schedule your treatment sessions. We will also provide you a safety card with information on whom to contact should you have any health concerns during the study. We will make sure these details are on your mobile phone also.

Confirmation of enrolment: Once we have the results of your blood test back, we will contact you to confirm your enrolment and your next session time.

Treatment session 1: We will conduct a quick health check and then you will complete some questionnaires and a brief intake interview. Following this, you will receive your first microdose of either LSD or a placebo. The dose is taken from a vial using an oral syringe (this does not involve a needle; the dose is squirted underneath your tongue). This will be done under the clinical supervision of study staff, and your health, heart rate and blood pressure will be checked. We will also ask you to complete some questionnaires during this time. You will then take part in an hour-long session of meaning-centred psychotherapy with a registered psychologist. Following this session, we will help you to install the study app onto your phone and show you how to use both the app and the activity tracker. Finally, we

will conduct a short discharge interview before you leave for the day. The length of this first treatment session will be approximately 7 hours.

We ask that you do not drive yourself to the clinic on this day and instead arrange to be picked up by a friend, otherwise we can arrange a taxi for you.

At this session, you will be given a four-week supply of the drug for you to take yourself at home. You will take the drug once each week at home before returning for the following week's treatment session. We will send you notifications in the study app to remind you. The study app will provide clear instructions for dosing at-home, we will also provide training during your first session.

Treatment sessions 2-7: The following week you will return for your second session of meaning-centred psychotherapy. This visit will last approximately 2 hours. This visit (and the next six) will follow the same structure. You will receive a re-supply of the last two at-home doses during treatment session four. The last visit will be 43 days after your first session.

Follow-up: One month and six months following the treatment sessions completion, we will contact you for a follow-up interview to check on your health. This will take approximately 30 minutes. We will also ask you to complete some questionnaires and ask you some questions about your experience in the study.

Following completion of the trial we will contact you again to debrief you and let you know whether you received the active dose of LSD or one of the placebos. This may be up to 2 years after your first treatment session. We understand this is a long time, but it is important that, for scientific purposes, our researchers remain unaware what condition each participant has been randomised to complete.

Study app and activity tracker: As a part of this research, our study team has developed a mobile phone application designed to make it easier for you to complete study tasks. This app will provide reminders for home dosing and upcoming treatment sessions, enable you to log when you have taken the dose, provide guidance on study procedures, and complete a few short questions on your dosing days. A Garmin activity tracker, will record simple things like heart rate and your physical activity. As above, training for using the app and activity tracker will be carried out at your first treatment session. The activity tracker will then be returned to study staff at your last treatment visit. The app can be uninstalled once we have completed the one-month follow-up phone call. Wecan assist you with that. More details on this are provided below.

Study Timeline	What will happen?	Approximate Duration	When
**T0 Visit 1 - Screening**	Medical history Questionnaires<br>Medical exam/questions<br>ECG<br>Blood samples	2 hours	Week 0
**T1 Visit 2 – Treatment session 1 and Dose 1**	Medical exam/questions<br>Qualitative Interview<br>Drug dose (could be LSD or	7 hours	Week 1

	placebo)<br>Questionnaires<br>Whānau Questionnaire and Interview<br>MCP Session<br>Discharge interview		
	Dose 2 at home		Week 1
**T2 Visit 3 – Treatment session 2 and Dose 3**	Medical exam/questions<br>Drug dose (could be LSD or placebo)<br>Questionnaires<br>MCP Session<br>Discharge interview	2 hours	Week 2
	Dose 4 at home		Week 2
**T3 Visit 4 – Treatment session 3 and Dose 5**	Medical exam/questions<br>Drug dose (could be LSD or placebo)<br>Questionnaires<br>MCP Session<br>Discharge interview	2 hours	Week 3
	Dose 6 at home		Week 3
**T4 Visit 5 – Treatment session 4 and Dose 7**	Medical exam/questions<br>Drug dose (could be LSD or placebo)<br>Questionnaires<br>MCP Session<br>Discharge interview	2.5 hours	Week 4
	Dose 8 at home		Week 4
**T5 Visit 6 – Treatment session 5 and Dose 9**	Medical exam/questions<br>Drug dose (could be LSD or placebo)<br>Questionnaires<br>MCP Session<br>Discharge interview	2 hours	Week 5
	Dose 10 at home		Week 5
**T6 Visit 7 – Treatment session 6 and Dose 11**	Medical exam/questions<br>Drug dose (could be LSD or placebo)<br>Questionnaires<br>MCP Session<br>Discharge interview	2 hours	Week 6
	Dose 12 at home		Week 6
**T7 Visit 8 – Treatment session 7 and Dose 13**	Medical exam/questions<br>Drug dose (could be LSD or placebo)<br>Questionnaires<br>Whānau Questionnaire<br>MCP Session	3 hours	Week 7

	ECG<br>Discharge interview		
**1-month follow-up**	Short follow-up interview Whānau follow-up interview	30 mins	Approx. 3 months since study start
**6-month follow-up**	Short follow-up interview	30 mins	Approx. 9 months since study start

WHAT WILL HAPPEN TO MY BLOOD SAMPLES?

Blood samples will be collected only once during the screening session. These samples will be sent to LabPlus for analysis. We will be looking at simple health markers (e.g. liver and kidney function) to ensure participants are safe to be involved in the research. If you are sexually active and of child-bearing potential (able to become pregnant), it is very important that you are not pregnant or become pregnant during this study. As such, LabPlus will also conduct a blood pregnancy test. For further information about the study requirements surrounding pregnancy, see below (possible risks of this study).

Only LabPlus will have access to these samples, The samples will be disposed of in accordance with LabPlus policy after they have been analysed. We will inform you of any unexpected results from these tests and suggest an appropriate course of action.

We acknowledge that personal and health information is a tāonga (treasure) and will be treated accordingly. We will not retain any samples and will ensure culturally appropriate processes regarding data management including maintaining privacy. No blood samples will leave Aotearoa.

WHAT ARE THE POSSIBLE RISKS OF THIS STUDY?

In the event that a condition which is assessed to be a clinical abnormality is detected through the blood test, you will be informed. Your general practitioner or other health professional of your choice will be notified.

The drug that we will use in this study is given at very low doses. We will be very careful and your first dose will be given under clinical supervision. If you receive LSD, this has been prepared by a pharmaceutical company under strict manufacturing conditions to ensure the drug is pure.

Previous studies of LSD have found some participants reported mild/moderate intensity headaches, anxiety and jitteriness. You might also experience these. If you do, it is important to let us know so we can advise you. Some participants also reported feeling some stimulation, a bit like you feel after drinking coffee. If you experience this, we have a plan in place to change the dose or amount of the substance you receive. The amount you receive at every dose will change every day – called a ‘titration protocol’. Trial staff will guide you through how this works and the study app will also assist with this procedure.

Although the doses of the drug given are very low and should not have any psychedelic effects, we advise that you should not drive, operate machinery, make any major decisions or engage in any dangerous activities or important care giving roles where you could put yourself or others in harm's way for six hours after taking a dose. Do not change these plans, even if you do not feel any effects or have not felt any effects so far. Any dose that you receive could contain LSD or a placebo and your reaction to each dose might not be the same, so it is essential that you maintain these arrangements for every dose day.

You should be aware that LSD is a Class A controlled substance. Although given at very low doses, taking this regularly may be incompatible with your employment conditions. You should discuss this with your employer and/or the study team before starting the study.

It is important that you keep the study drugs safe, out of the reach of children and you may not share them with others. If required, we can provide you with a lockbox to keep your home doses safe. You should take them exactly as requested. It may be unsafe for you to not do so. Please discuss this with the study team.

You should be aware that the LSD being given to you will not be available to you after the study is finished. LSD is an illegal Class A drug in New Zealand.

For participants currently taking antidepressant medication, the interaction between psychedelics and antidepressants is currently unknown. Recent evidence looking at this interaction effect suggests there may be minimal effects, however this type of research is still in its early stages. It is theoretically possible that LSD and antidepressants used together might cause "serotonin syndrome" which can cause shivering, fever, muscle rigidity and even seizures. As this study involves taking very small doses of LSD we do not expect to see any negative interactions. Your safety for participation will be established by our study physician at screening. We also have a number of safety protocols in place to check and monitor your response. If you have any questions about this, please feel free to speak with your doctor or with study staff. We are happy to answer any questions you might have.

As a part of this research, we will ask you to complete a number of psychological questionnaires. These include questionnaires that ask about depression and suicide. If these cause you distress our research team is available to support you or refer you to someone else who can.

Reproductive Risks for Sexually Active Participants of Child-Bearing Potential

The effects of LSD in pregnancy and breastfeeding are unknown, but there is a risk it may cause birth defects or fetal deaths, and/or be passed on in breast milk. If you are pregnant or breastfeeding, you cannot take part in this study.

If you are sexually active and of child-bearing potential (able to become pregnant), it is very important that you do not become pregnant during this study. You must use one of the methods of contraception listed below, from at least 10 days before your first dose of study drug until at least 72 hours after your last dose:

A highly effective method (less than 1 pregnancy per 100 women using the method for one year) e.g.:

- Implant contraceptive (e.g. Jadelle®)

- Intra-uterine device (IUD) containing either copper or levonorgestrel (e.g. Mirena®)
- Male sterilization (vasectomy)
- Female sterilization (e.g. bilateral tubal ligation ('clipping or tying tubes') or hysterectomy)

OR an effective method (5 - 10 pregnancies per 100 women using the method for one year) e.g.:

- Injectable contraceptive (e.g. Depo Provera)
- Oral Contraceptive Pill (combined hormonal contraceptive pill or progestogen-only 'mini-pill')
- Vaginal contraceptive ring (e.g. NuvaRing®)

You must also agree not to donate eggs, from dosing until at least 3 months after your last dose of study drug.

If you do become pregnant during the study, you must tell the study doctor as soon as possible. If you are pregnant, this will result in the cessation of the study for you and we will ask to collect information about the pregnancy and outcomes, including that of the infant.

Reproductive Risks for Sexually Active Participants able to Father a Child.

The effects of LSD if passed on through semen are unknown, but there is a risk it may cause birth defects or fetal deaths. **You are responsible for informing your sexual partner** of these possible risks.

If you are sexually active and have any partner who is of child-bearing potential (meaning a partner who may become pregnant) it is very important that you use contraception during this study. You and your partner must use one of the contraception options listed above for participants of child-bearing potential, from at least 10 days before your first dose of study drug through until at least 72 hours after your last dose.

If a pregnancy occurs, you must report this to a member of the study team as soon as possible. Your partner will be asked to give consent for her information and her infant's information to be collected for monitoring purposes.

You must also agree not to donate sperm, from dosing until at least 3 months after your last dose of the study drug.

WHAT ARE THE POSSIBLE BENEFITS OF THIS STUDY?

Although there may be no direct benefits to you in taking part in this study, meaning-centred psychotherapy is a validated talk therapy used to manage psychological distress in cancer patients and previous research has shown benefits from receiving this form of psychotherapy. Given that everyone will receive meaning-centred psychotherapy, we hope that all participants will receive some benefit.

WHAT ARE THE ALTERNATIVES TO TAKING PART?

You do not have to participate in this study to receive treatment for any cancer-related distress you may be experiencing. There are other treatments available that are known to be effective. These can be discussed with your doctor.

Will any costs be reimbursed?

We will reimburse you with vouchers for any reasonable travel and food costs relating to the study. If you require a taxi to get to and from the study then we can arrange and pay for this. Please keep any receipts so that we can give you vouchers to reimburse you for your costs. We recognise that taking part in the study will take **up to 25 hours** of your time and several months of contact with us and we will provide you with $200 vouchers at the end of the 1-month follow-up interview in recognition of this inconvenience.

What if something goes wrong?

As this research study is for the principal benefit of its commercial sponsor The University of Auckland, if you are injured as a result of taking part in this study, you **will not** be eligible for compensation from ACC.

However, The University of Auckland has satisfied the Southern Health and Disability Ethics Committee that approved this study that it has up-to-date insurance for providing participants with compensation if they are injured as a result of taking part in this study.

New Zealand ethical standards require compensation for injury to be at least ACC equivalent. Compensation should be appropriate to the nature, severity and persistence of your injury and should be no less than would be awarded for similar injuries by New Zealand's ACC scheme.

Some sponsors voluntarily commit to providing compensation in accordance with guidelines that they have agreed between themselves, called the Medicines New Zealand Guidelines (Industry Guidelines). These are often referred to for information on compensation for commercial clinical trials. There are some important points to know about the Industry Guidelines:

- On their own they are not legally enforceable and may not provide ACC equivalent compensation.
- There are limitations to when compensation is available, for example, compensation may be available for more serious, enduring injuries, and not for temporary pain or discomfort or less serious or curable complaints.

Unlike ACC, the guidelines do not provide compensation on a no-fault basis, so the Sponsor may not accept the compensation claim if:

- Your injury was caused by the investigators, or
- There was a deviation from the proposed research plan, or
- Your injury was caused solely by you.

An initial decision whether to compensate you would be made by the sponsor and/or its insurers.

If they decide not to compensate you, you may be able to take action through the Courts for compensation, but it could be expensive and lengthy, and you might require legal representation. You would need to be able to show that your injury was caused by participation in the trial.

You are strongly advised to read the Industry Guidelines and ask questions if you are unsure about what they mean for you.

If you have private health or life insurance, you may wish to check with your insurer that taking part in this study won't affect your cover.

WHAT WILL HAPPEN TO MY INFORMATION?

During this study, the study doctors/researchers, nurses and other study staff will record information about you and your study participation. This includes the results of any study assessments. If needed, information from your hospital records and your GP may also be collected; access to your records is limited to that required for study purposes. As part of this research, we will need to video record the psychotherapy sessions to ensure that the therapy is delivered as it should be. You cannot take part in this study if you do not consent to the collection of this information.

Identifiable Information

Identifiable information is any data that could identify you (e.g. your name, date of birth, or address). The following groups may have access to your identifiable information:

- University of Auckland investigators, staff and PhD and Masters students (to complete study assessments).
- LabPlus staff, to process and report your screening blood tests.
- The University of Auckland (the sponsor) and/or the Health Research Council (the funder) and its representatives, if you make a compensation claim for study-related injury. Identifiable information is required in order to assess your claim.
- The University of Auckland, the funder, ethics committees, or government agencies from New Zealand, if the study or site is audited. Audits are done to make sure that participants are protected, the study is run properly, and the data collected is correct.
- Your usual doctor, if a study test gives an unexpected result that could be important for your health. This allows appropriate follow-up to be arranged.
- Rarely, it may be necessary for a Study Doctor to share your information with other people – for example, if there is a serious threat to public health or safety, or to the life or health of you or another person OR if the information is required in certain legal situations.

De-identified (Coded) Information

To make sure your personal information is kept confidential, information that identifies you will not be included in any report generated by the study team and any study information sent to the sponsor and/or funder. Instead, you will be identified by a code. The study team will keep a list linking your code with your name, so that you can be identified by your coded data if needed.

The following groups may have access to your coded information:

- The sponsor, for the purposes of this study.
- International and National collaborators / companies / researchers.
- Ethics committees, health, regulatory or other governmental agencies.

The results of the study may be published or presented, but not in a form that would reasonably be expected to identify you.

Anonymised Information.

The sponsor may remove the code from your de-identified information – this is called 'anonymisation'. This makes it very difficult (but not impossible) to identify the information that belongs to you. The sponsor may use this information for future research (see below).

Future Research Using Your Information.

Your coded information may be used for future research related to microdosing LSD or cancer. Your coded information may also be used for other medical and/or scientific research that is unrelated to the current study. This does not include your tissue samples, which will be disposed after they are analysed.

This future research may be conducted overseas. You will not be told when future research is undertaken using your information. Your information may be shared widely with other researchers or companies. Your information may also be added to information from other studies, to form much larger sets of data.

You will not get reports or other information about any research that is done using your information.

Your information may be used indefinitely for future research unless you withdraw your consent. However, it may be extremely difficult or impossible to access your information, or withdraw consent for its use, once your information has been shared for future research.

Security and Storage of Your Information.

Your identifiable information is held at The University of Auckland during the study. After the study it is transferred to a secure archiving site and stored for at least 15 years, then destroyed. Your coded information will be entered into electronic case report forms and sent through a secure server to the sponsor. Coded study information will be kept by the sponsor in secure, cloud-based storage indefinitely. All storage will comply with local and/or international data security guidelines. Your video data will be kept in password encrypted files for additional security.

Risks.

Although efforts will be made to protect your privacy, absolute confidentiality of your information cannot be guaranteed. Even with coded and anonymised information, there is no guarantee that you cannot be identified. The risk of people accessing and misusing your information (e.g. making it harder for you to get or keep a job or health insurance) is currently very small but may increase in the future as people find new ways of tracing information.

Your coded / anonymised information may be sent overseas. Other countries may have lower levels of data protection than New Zealand. There may be no New Zealand representation on overseas organisations which make decisions about the use of your information. There is a risk that overseas researchers may work with information in a way that is not culturally appropriate for New Zealanders.

Rights to Access Your Information.

You have the right to request access to your information held by the research team. You also have the right to request that any information you disagree with is corrected.

Please ask if you would like to access the results of your screening and safety tests during the study, however this could result in you being withdrawn from the study to protect the study's scientific integrity.

If you have any questions about the collection and use of information about you, you should ask the study team.

Rights to Withdraw Your Information.

You may withdraw your consent for the collection and use of your information at any time, by informing a member of the study team.

If you withdraw your consent, your study participation will end, and the study team will stop collecting information from you.

Information collected up until your withdrawal from the study will continue to be used and included in the study. This is to protect the quality of the study.

Ownership Rights.

Information from this study may lead to discoveries and inventions or the development of a commercial product. The rights to these will belong to the sponsor or the funder. You and your family will not receive any financial benefits or compensation, nor have any rights in any developments, inventions, or other discoveries that might come from this information.

Use of New Technologies (Mobile phone App and Activity Tracking)

In this study use of a mobile phone application and Garmin activity tracker are mandatory components for study participation. We will provide you with the Activity tracker and if you do not have a mobile phone or data plan we will provide you with one or both as needed.

The mobile phone app has been written by researchers at The University of Auckland. It is designed to collect questionnaire data from you on dosing days, has a calendar function to help remind you of visits, help you log adverse effects and provide other information about the study for you so you can access it easily. All the information collected via the mobile phone app is de-identified. We will use a study identifier in the app and no personally identifiable information will be held on the app. The app will send data directly to our study database where the rest of your data is held.

We will ask you to wear a Garmin Activity tracker for the duration of the study as we are interested in recording your activity and body functions during the study. These data will be sent to the Garmin website and to an associated company called Fitrockr.

In order to protect your privacy we will make dummy email addresses and accounts to associate with the watch that you will wear so that your personal information does not need to be registered with Garmin/Fitrockr. Before we give you the watch, we will turn the GPS function off so that neither the research team nor anyone else can track your location. We would ask you not to turn the GPS functions on. Only the research team and you have access to your dummy account and password.

Although your data is de-identified you should note that your data will be sorted overseas in both the United States for Garmin and in Germany for FitRockr. These companies may use your de-identified data for their own purposes beyond the aims of our study. Although these

companies require consent to share your de-identified data with third parties we are unable to guarantee this is the case.

There are no costs to you involved with using the watch and its services. We will need to install the Garmin app on your phone so that your data can be transmitted from the watch to your phone to the Garmin/Fitrockr overseas data clouds. We will access your data from there. We will ask you to return the Activity tracker to us at the end of the study.

The full data policies of these companies are at these links:

https://www.fitrockr.com/health-solutions/privacy-policy/

https://www.garmin.com/en-NZ/privacy/connect/policy/#categoriesOfPersonalDataProcessedByGarmin

What are the risks?
We have designed this approach to protect your privacy and reduce the potential for any personal health information to be accidentally disclosed. However, because information is being transmitted over the internet, there is still some risk of accidental disclosure of your de-identified information.

What happens after the study or if I change my mind?

The LSD that you receive during the study will not be available to you after your participation in this study, as it is an illegal Class A drug in New Zealand. However, meaning-centred psychotherapy is an established form of therapy in New Zealand, and you can choose to seek out continued treatment if you wish. Please talk to your doctor about this.

You can be informed which treatment group you were allocated to only after the final participant has completed the study. This may be more than two years after you complete the study if you were one of the first participants.

You may withdraw your consent for the collection and use of your information at any time, by informing a member of the study team. If you withdraw your consent, your study participation will end, and the study team will stop collecting information from you. Information collected up until your withdrawal from the study will continue to be used and included in the study. This is to protect the quality of the study.

Can I find out the results of the study?

It can take quite a long time to analyse data from these kinds of studies. We hope to be able to tell you the final results one to two years after completion of the study. We plan to publish the results in specialised academic journals. If you want us to, we can send you a summary of the results in an easier format to read.

This trial is registered on the Australian New Zealand Clinical Trials Registry (ANZCTR). This can be accessed at anzctr.org.au

Who is funding the study?

The Health Research Council (HRC) of New Zealand and MindBio Therapeutics Ltd.

WHO HAS APPROVED THE STUDY?

This study has been approved by an independent group of people called a Health and Disability Ethics Committee (HDEC), who check that studies meet established ethical standards. The Southern Health and Disability Ethics Committee has approved this study.

The scientific aspects of this study have been approved by the Standing Committee on Therapeutic Trials (SCOTT), which is part of Medsafe.

WHO DO I CONTACT FOR MORE INFORMATION OR IF I HAVE CONCERNS?

If you have any questions, concerns or complaints about the study at any stage, you can contact:

Dr Lisa Reynolds, Senior Lecturer
Phone: 09 923 4938
Email: l.reynolds@auckland.ac.nz

If you want to talk to someone who isn't involved with the study, you can contact an independent health and disability advocate on:

Phone:	0800 555 050
Fax:	0800 2 SUPPORT (0800 2787 7678)
Email:	advocacy@advocacy.org.nz
Website:	https://www.advocacy.org.nz/

For Maori health support please contact:

He Kamaka Waiora (Maori Health Team)
Telephone number: 09 486 8324 x 2324
Email: hkw@adhb.govt.nz

You can also contact the health and disability ethics committee (HDEC) that approved this study on:

Phone:	0800 4 ETHIC
Email:	hdecs@health.govt.nz

Consent Form

Feasibility study of Psychedelic Microdosing-Assisted Meaning Centred Psychotherapy in advanced stage cancer patients

Please tick to indicate you consent to the following

I have read and I understand the Participant Information Sheet.	
I have been given sufficient time to consider whether or not to participate in this study.	
I have had the opportunity to use a legal representative, whānau/ family support or a friend to help me ask questions and understand the study.	
I am satisfied with the answers I have been given regarding the study and I have a copy of this consent form and information sheet.	
I understand that taking part in this study is voluntary (my choice) and that I may withdraw from the study at any time without this affecting my medical care.	
I consent to the research staff collecting and processing my information, including information about my health.	
I consent to my information being sent overseas.	
If I decide to withdraw from the study, I agree that the information collected about me up to the point when I withdraw may continue to be processed.	
I consent to my GP or current provider being informed about my participation in the study and of any significant abnormal results obtained during the study.	
I understand that there may be risks associated with the treatment in the event of myself or my partner becoming pregnant. I undertake to inform my partner of the risks and to take responsibility for the prevention of pregnancy.	
I agree to my blood samples being collected and I am aware that these samples will be disposed of using established guidelines for discarding biohazard waste.	
I agree to an approved auditor appointed by the New Zealand Health and Disability Ethics Committees, or any relevant regulatory authority or their approved representative reviewing my relevant medical records for the sole purpose of checking the accuracy of the information recorded for the study.	
I understand that my participation in this study is confidential and	

that no material, which could identify me personally, will be used in any reports on this study.	
I understand the compensation provisions in case of injury during the study.	
I know who to contact if I have any questions about the study in general.	
I understand that LSD is a Class A controlled substance and remains the property of the University of Auckland and must be returned if requested and must only be used as directed. I understand that failure to do these things may be a criminal offence and the New Zealand police may be contacted	
I will take the drugs as directed, will keep them out of reach of children and will not provide them to any other persons and not engage in dangerous activities (like driving) for six hours after taking drug doses.	
I understand my responsibilities as a study participant.	
I wish to receive a summary of the results from the study.	Yes ☐ No ☐

Declaration by participant:
I hereby consent to take part in this study.

Participant's name: ______________________

Signature: ______________________ Date: ______________________

Declaration by member of research team:

I have given a verbal explanation of the research project to the participant and have answered the participant's questions about it.

I believe that the participant understands the study and has given informed consent to participate.

Researcher's name: ______________________

Signature: ______________________ Date: ______________________
