# Supplementary material for: PAM trial protocol: a randomised feasibility study of psychedelic microdosing–assisted meaning-centred psychotherapy in advanced stage cancer patients
Source: Pilot Feasibility Stud. 2024 Feb 12;10:29. doi: 10.1186/s40814-024-01449-9 (PMC10860284; doi:10.1186/s40814-024-01449-9)
Supplement: Supplementary file 2 — Additional file 2. [file 40814_2024_1449_MOESM2_ESM.docx]

Figure 1 Schedule of enrolment, interventions, and assessments (full)

|  | **STUDY PERIOD** | | | | | | | | | | | | | | | | | | | |  |  |
| --- | --- | --- | --- | --- | --- | --- | --- | --- | --- | --- | --- | --- | --- | --- | --- | --- | --- | --- | --- | --- | --- | --- |
|  | **Screening** | **Confirm Enrolment** | **Treatment** |  |  |  |  |  | **HD** | **T2** | **HD** | **T3** | **HD** | **T4** | **HD** | **T5** | **HD** | **T6** | **HD** | **T7** | **Follow up 1** | **Follow up 2** |
| **TIMEPOINT** | **Day -14 to -1** | **Day -12 to -1** | **Day 1 Baseline** | **30 mins** | **60 mins** | **120 mins** | **240 mins** | **360 mins** | **Day 4** | **Day 8** | **Day 11** | **Day 15** | **Day 18** | **Day 22** | **Day 25** | **Day 29** | **Day 32** | **Day 36** | **Day 39** | **Day 43** | **1 month** | **6 months** |
| **Enrolment:** |  |  |  |  |  |  |  |  |  |  |  |  |  |  |  |  |  |  |  |  |  |  |
| Eligibility screen | X |  |  |  |  |  |  |  |  |  |  |  |  |  |  |  |  |  |  |  |  |  |
| Informed consent | X |  |  |  |  |  |  |  |  |  |  |  |  |  |  |  |  |  |  |  |  |  |
| Randomization |  | X |  |  |  |  |  |  |  |  |  |  |  |  |  |  |  |  |  |  |  |  |
| **Interventions:** |  |  |  |  |  |  |  |  |  |  |  |  |  |  |  |  |  |  |  |  |  |  |
| LSD 8µg (4-20µg) or placebo |  |  | X |  |  |  |  |  | X |  |  |  |  |  |  |  |  |  |  | X |  |  |
| MCP |  |  |  |  | X |  |  |  |  | X |  | X |  | X |  | X |  | X |  | X |  |  |
| **ASSESSMENTS** |  |  |  |  |  |  |  |  |  |  |  |  |  |  |  |  |  |  |  |  |  |  |
| **Psychiatric:** |  |  |  |  |  |  |  |  |  |  |  |  |  |  |  |  |  |  |  |  |  |  |
| Psychiatric Interview | X |  |  |  |  |  |  |  |  |  |  |  |  |  |  |  |  |  |  |  |  |  |
| Mini | X |  |  |  |  |  |  |  |  |  |  |  |  |  |  |  |  |  |  |  |  |  |
| Mini-Ace | X |  |  |  |  |  |  |  |  |  |  |  |  |  |  |  |  |  |  |  |  |  |
| C-SSRS | X |  | X |  |  |  |  |  |  | X |  | X |  | X |  | X |  | X |  | X |  |  |
| **Physiological:** |  |  |  |  |  |  |  |  |  |  |  |  |  |  |  |  |  |  |  |  |  |  |
| Medical History | X |  |  |  |  |  |  |  |  |  |  |  |  |  |  |  |  |  |  |  |  |  |
| Height | X |  |  |  |  |  |  |  |  |  |  |  |  |  |  |  |  |  |  |  |  |  |
| Weight | X |  |  |  |  |  |  |  |  |  |  |  |  |  |  |  |  |  |  |  |  |  |
| AKPS | X |  | X |  |  |  |  |  |  | X |  | X |  | X |  | X |  | X |  | X |  |  |
| Medication | X |  | X |  |  |  |  |  |  | X |  | X |  | X |  | X |  | X |  | X |  |  |
| Health status review | X |  | X |  |  |  |  |  |  | X |  | X |  | X |  | X |  | X |  | X |  |  |
| Vital signs | X |  | X | X |  | X | X | X |  | X |  | X |  | X |  | X |  | X |  | X |  |  |
| Serotonin Syndrome checks | X |  | X | X |  | X | X | X |  | X |  | X |  | X |  | X |  | X |  | X |  |  |
| Blood test | X |  |  |  |  |  |  |  |  |  |  |  |  |  |  |  |  |  |  |  |  |  |
| Adverse events |  |  | X |  |  |  |  |  |  |  |  |  |  |  |  |  |  |  |  |  | X |  |
| Sleep and activity tracker |  |  | X |  |  |  |  |  |  |  |  |  |  |  |  |  |  |  |  |  | X |  |
| ECG | X |  |  |  |  |  |  |  |  |  |  |  |  |  |  |  |  |  |  | X |  |  |
| PCOC-SAS |  |  | X |  |  |  |  |  |  | X |  | X |  | X |  | X |  | X |  | X |  |  |
| BPI-SF |  |  | X |  |  |  |  |  |  |  |  |  |  | X |  |  |  |  |  | X | X | X |
| **Psychological:** |  |  |  |  |  |  |  |  |  |  |  |  |  |  |  |  |  |  |  |  |  |  |
| DASS-21 | X |  |  |  |  |  |  |  |  |  |  |  |  | X |  |  |  |  |  | X | X | X |
| HADS | X |  |  |  |  |  |  |  |  |  |  |  |  | X |  |  |  |  |  | X | X | X |
| MODTAS | X |  |  |  |  |  |  |  |  |  |  |  |  |  |  |  |  |  |  |  |  |  |
| TEX-Q |  |  | X |  |  |  |  |  |  |  |  |  |  |  |  |  |  |  |  | X |  |  |
| FACT-G |  |  | X |  |  |  |  |  |  |  |  |  |  | X |  |  |  |  |  | X | X | X |
| LAP-R-PMI |  |  | X |  |  |  |  |  |  |  |  |  |  | X |  |  |  |  |  | X | X | X |
| FACIT-SP-12 |  |  | X |  |  |  |  |  |  |  |  |  |  | X |  |  |  |  |  | X | X | X |
| Demoralization Scale |  |  | X |  |  |  |  |  |  |  |  |  |  | X |  |  |  |  |  | X | X | X |
| SAHD |  |  | X |  |  |  |  |  |  |  |  |  |  | X |  |  |  |  |  | X | X | X |
| WCS |  |  | X |  |  |  |  |  |  |  |  |  |  | X |  |  |  |  |  | X | X | X |
| BFI-2-XS | X |  |  |  |  |  |  |  |  |  |  |  |  | X |  |  |  |  |  | X | X | X |
| 5D-ASC |  |  |  |  |  |  |  | X |  |  |  |  |  |  |  |  |  |  |  |  |  |  |
| Hua Oranga – Participant |  |  | X |  |  |  |  |  |  |  |  |  |  |  |  |  |  |  |  | X |  |  |
| Hua Oranga – whanau |  |  | X |  |  |  |  |  |  |  |  |  |  |  |  |  |  |  |  | X |  |  |
| Hua Oranga – clinician |  |  |  |  |  | X |  |  |  |  |  |  |  |  |  |  |  |  |  | X |  |  |
| WAI-SF – Participant |  |  |  |  |  | X |  |  |  |  |  |  |  |  |  |  |  |  |  | X |  |  |
| WAI-SF – clinician |  |  |  |  |  | X |  |  |  |  |  |  |  |  |  |  |  |  |  | X |  |  |
| Blinding |  |  |  |  |  |  |  | X |  |  |  |  |  |  |  |  |  |  |  | X |  |  |
| **Qualitative:** |  |  |  |  |  |  |  |  |  |  |  |  |  |  |  |  |  |  |  |  |  |  |
| Subject release interview |  |  |  |  |  |  |  | X |  | X |  | X |  | X |  | X |  | X |  | X |  |  |
| Telephone follow-up |  |  |  |  |  |  |  |  |  |  |  |  |  |  |  |  |  |  |  |  | X | X |

NB: HD = Home dosing
